# Monte Carlo‐based investigation of absorbed‐dose energy dependence of radiochromic films in high energy brachytherapy dosimetry

**DOI:** 10.1120/jacmp.v15i1.4448

**Published:** 2014-01-06

**Authors:** Mishra Subhalaxmi, T. Palani Selvam

**Affiliations:** ^1^ Radiological Physics & Advisory Division Health, Safety & Environment Group, Bhabha Atomic Research Centre Mumbai 400 094 Maharashtra India

**Keywords:** brachytherapy, Monte Carlo, energy response, phantoms

## Abstract

Relative absorbed dose energy response correction, R, for various radiochromic films in water phantom is calculated by the use of the Monte Carlo‐based EGSnrc code system for high energy brachytherapy sources ${}^{60}\text{Co}$, ${}^{137}\text{Cs}$, ${}^{192}\text{Ir}$ and ${}^{169}\text{Yb}$. The corrections are calculated along the transverse axis of the sources (1‐15 cm). The radiochromic films investigated are EBT, EBT2 (lot 020609 and lot 031109), RTQA, XRT, XRQA, and HS. For the ${}^{60}\text{Co}$ source, the value of R is about unity and is independent of distance in the water phantom for films other than XRT and XRQA. The XRT and XRQA films showed distance‐dependent R values for this source (the values of R at 15 cm from the source in water are 1.845 and 2.495 for the films XRT and XRQA, respectively). In the case of ${}^{137}\text{Cs}$ and ${}^{192}\text{Ir}$ sources, XRT, XRQA, EBT2 (lot 031109), and HS films showed distant‐dependent R values. The rest of the films showed no energy dependence (HS film showed R values less than unity by about 5%, whereas the other films showed R values higher than unity). In the case of ${}^{169}\text{Yb}$ source, the EBT film showed no energy dependence and EBT2 film (lot 031109) showed a distance‐independent R value of 1.041. The rest of the films showed distance‐dependent R values (increases with distance for the films other than HS). The solid phantoms PMMA and polystyrene enhance the R values for some films when compared the same in the water phantom.

PACS number: 87.53.Jw

## I. INTRODUCTION

Accurate dose measurement in the vicinity of brachytherapy source is difficult mainly due to existence of steep dose gradients.^1^, ^2^ Hence requirements of a suitable dosimeter for measuring accurate dose in vicinity of brachytherapy source are high spatial resolution, energy independency, tissue equivalency, and convenience of use. The introduction of radiochromic films has solved some of the problems associated with conventional 2D radiation detectors such as ionization chambers, thermoluminescence dosimetry (TLD), diodes, plastic scintillators, diamond detectors, radiographic films, and polymer gels. The high spatial resolution with small detecting volume makes them suitable for measurement of dose distributions in radiation fields with highdose gradients. Radiochromic films change color directly upon irradiation; hence, they do not require chemical processing. These dosimeters are insensitive to visible light, and thus can be handled in room light.^3^, ^4^, ^5^ Radiochromic films are in use extensively for radiation dosimetry in conventional radiation therapy, including external‐beam, brachytherapy, and radiosurgery.^6^, ^7^, ^8^, ^9^, ^10^, ^11^, ^12^, ^13^ Varieties of radiochromic films are commercially available and, depending upon the type of application, user can chose a suitable radiochromic film. Some films, such as XRT and XRQA, contain high‐Z materials in the emulsion and are designed for use in the kilo‐voltage range, whereas film HS, which contains low‐Z material in the emulsion, is designed for measurement of absorbed dose in high‐energy photon beams (above 1 MeV).^(14)^

In a previously published study, Monte Carlo‐based relative absorbed‐dose energy response correction as a function of depth in water was investigated for solid state detectors such as LiF, ${\text{Li}}_{2}B_{4}O_{7}$, Si diode, diamond, ${\text{Al}}_{2}O_{3}$ for ${}^{125}I$ and ${}^{169}\text{Yb}$ brachytherapy sources.^(15)^ Influence of solid phantoms, such as polystyrene and polymethyl methacrylate (PMMA), was also investigated in the work. The present study is aimed at calculating relative absorbed‐dose energy response correction as a function of depth in water for the various radiochromic films for different brachytherapy sources such as ${}^{60}\text{Co}$, ${}^{137}\text{Cs}$, ${}^{192}\text{Ir}$, and ${}^{169}\text{Yb}$. This study also includes the influence of solid phantoms polystyrene and PMMA on the correction. We have employed the Monte Carlo‐based user codes DOSRZnrc and FLURZnrc^(16)^ of the EGSnrc code system^(17)^ in the present work.

## II. MATERIALS AND METHODS

### A. Radioactive sources and radiochromic films

The brachytherapy sources investigated in this study are BEBIG high‐dose rate (HDR) ${}^{60}\text{Co}$ (model Co0.A86; Eckert & Zielger BEBIG GmbH, Berlin, Germany),^(18)^ ${}^{137}\text{Cs}$ (model RTR; Radiation Therapy Resources, Valencia, CA)^(19)^ HDR ${}^{192}\text{Ir}$ (model MicroSelectron; Elekta, Stockholm, Sweden),^(20)^ and HDR ${}^{169}\text{Yb}$ (model 4140; Implant Sciences Corp., Wilmington, MA).^(21)^ The photon energy spectra of ${}^{169}\text{Yb}$ and ${}^{192}\text{Ir}$ needed for the Monte Carlo calculations are taken from literature.^21^, ^22^ For the ${}^{60}\text{Co}$ source, two gamma lines 1.17 MeV and 1.33 MeV are considered. For the ${}^{137}\text{Cs}$ source, photon of energy 0.662 MeV is used. Our investigation includes the radiochromic film models HS,^14^, ^23^ EBT,^(24)^ EBT2 (lot 031109),^(24)^ RTQA,^(25)^ EBT2 (lot 020609),^(24)^ XRT,^14^, ^23^ and XRQA.^(26)^ Above films are listed in order of their increasing atomic number (Z). The composition and structural details of these investigated films are taken from the published studies. Table 1 presents the composition, $Z_{\text{eff}}$ (effective atomic number), <Z/A> (electron density), and p (mass density), and Table 2 presents the structural details of the investigated films.

**Table 1:** Elemental composition, effective atomic number $Z_{\text{eff}}$, electron density <Z/A>, and mass density ρ of the investigated radiochromic films and phantom materials

	*Composition (w eight fraction)*											
*Material*	*H*	*C*	*N*	*O*	*Li*	*Cl*	*K*	*Br*	*Cs*	$Z_{\text{eff}}$	<Z/A>	$\rho \;(g/cm^{3})$
Water	0.112	–	–	0.888	–	–	–	–	–	7.42	0.555	1
PMMA	0.081	0.599	–	0.320	–	–	–	–	–	6.47	0.539	1.19
Polystyrene	0.077	0.923	–	–	–	–	–	–	–	5.7	0.538	1.06
Polyester	0.042	0.625	–	0.333	–	–	–	–	–	6.64	0.52	1.35
Adhesive	0.094	0.656	–	0.249	–	–	–	–	–	6.26	0.546	1.2
Surface	0.065	0.323	0.216	0.205	0.023	0.168	—	—	—	9.9	0.527	1.2
*Composition of Active Layers*												
EBT	0.094	0.574	0.132	0.164	0.008	0.029	–	–	–	7.06	0.545	1.1
EBT2 (lot 020609)	0.096	0.578	0.002	0.278	0.009	0.017	0.006	0.013	–	9.17	0.538	1.2
EBT2 (lot 031109)	0.095	0.597	0.002	0.261	0.009	0.023	0.013	–	–	7.44	0.539	1.2
RTQA	0.091	0.537	0.127	0.142	0.019	0.084	–	–	–	8.28	0.541	1.1
XRT	0.078	0.462	0.115	0.143	–	–	–	0.076	0.126	26.59	0.523	1.75
HS	0.090	0.570	0.160	0.180	–	–	–	–	–	6.28	0.544	1.08
XRQA	0.064	0.381	0.055	0.138	0.040	–	–	0.134	0.223	34.52	0.501	1.2

**Table 2:** Structural details of the investigated radiochromic films. All the dimensions are in μm

	*EBT*	*EBT2 (lot 020609 and lot 031109)*	*RTQA*	*XRT*	*HS*	*XRQA*
Polyester	97	50	97	97	97	97
Adhesive	–	25	12	–	–	–
Active	17	–	–	30	40	25
Surface	6	5	3	–	–	10
Active	17	30	17	–	–	25
Polyester	97	175	97	97	97	97

### B. Energy dependence of the detector

The energy dependence of a detector is composed of two parts: the intrinsic energy dependence (intrinsic beam quality dependence) and the absorbed‐dose energy dependence.

The intrinsic energy dependence, $k_{\text{bq}}(Q)$, is the ratio of the dose to the sensitive element of the detector at a given beam quality $D_{\text{det}}(Q)$ to the detector reading at the same beam quality $M_{\text{det}}(Q)$:

(1) $$D_{\text{det}}{(Q)=k}_{\text{bq}}{(Q)M}_{\text{det}}(Q)$$

The absorbed‐dose energy dependence, f(Q), is the ratio of the dose to the medium at the point of measurement of the detector in the absence of the detector, $D_{\text{med}}(Q)$, to the dose to the sensitive material of the detector, $D_{\text{det}}(Q)$:

(2) $$D_{\text{med}}(Q)=f(Q)D_{\text{det}}(Q)$$

In general, Monte Carlo simulation calculates only the absorbed‐dose energy dependence of a detector. It varies with different beam quality and also with the location of the detector for a given beam quality. The overall energy dependence of a detector, often referred to as the energy response, is the product of the intrinsic energy dependence and the absorbed‐dose energy dependence.

In brachytherapy, quantity of interest is dose to water. The detectors are generally calibrated against a reference beam, which is generally ${}^{60}\text{Co}$. For a given detector material and a beam quality Q, relative absorbed dose energy response correction factor R is defined as:

(3) $$R=\frac{{\left(D_{\text{det}}/D_{wat}\right)}_{Q}}{{\left(D_{\text{det}}/D_{wat}\right)}_{60_{Co}}}$$

where the numerator presents detector‐to‐water dose ratio at Q, and the denominator represents the same dose ratio at ${}^{60}\text{Co}$ beam energy.

In the presence of charged particle equilibrium, Eq. (3) can be rewritten as:

(4) $$R=\frac{\left[1/f(Q)\right]}{\left[1/f{\left(Q\right)}_{60_{Co}}\right]}=\frac{{\left[{\left(\mu_{en}/\rho \right)}_{\text{det}}/{\left(\mu_{en}/\rho \right)}_{wat}\right]}_{Q}}{{\left[{\left(\mu_{en}/\rho \right)}_{\text{det}}/{\left(\mu_{en}/\rho \right)}_{wat}\right]}_{60_{Co}}}$$

### C. Monte Carlo calculations

#### C.1 *DOSRZnrc simulations of dose ratios for* ${}^{60}\text{Co}$ *beam*

Film‐to‐water dose ratio, ${\left(\frac{D_{film}}{D_{wat}}\right)}_{60_{Co}}$, is calculated in the water phantom for each of the investigated films for ${}^{60}\text{Co}$ beam using the DOSRZnrc user code of EGSnrc code system. Here, $D_{\text{film}}$ and $D_{\text{wat}}$ represent the dose to active region of the film and dose to water, respectively. In the Monte Carlo calculation, a realistic ${}^{60}\text{Co}$ spectrum from a telecobalt unit is used. The ${}^{60}\text{Co}$ beam is parallel and has a radius of 5.64 cm at the front face of the phantom (equivalent field size is $10\times 10\;{\text{cm}}^{2}$). The beam is incident on a unity density cylindrical water phantom of 20 cm radius and 40 cm height. In the Monte Carlo calculations, the active layer of films is positioned at 0.5 cm depth along the central axis of the water phantom. All layers of the films are modeled as cylindrical discs with radius 0.5 cm. The thicknesses of the films are detailed in Table 2.

#### C.2 FLURZnrc simulations of collision kerma for brachytherapy sources

As described in the published work,^(15)^ calculation of dose ratio of film to water for the ${}^{60}\text{Co}$, ${}^{137}\text{Cs}$, ${}^{192}\text{Ir}$, and ${}^{169}\text{Yb}$ sources (numerator of Eq. (3)) is based on FLURZnrc user code.^(16)^ In the calculations, the photon fluence spectrum is scored in 0.5 mm thick and 0.5 mm high cylindrical shells, along the transverse axis of the sources (distances, 1 cm−15 cm) in 20 cm radius×40 cm high cylindrical phantoms (liquid water, PMMA, and polystyrene). The fluence spectrum is converted to collision kerma to water and collision kerma to films by using the mass energy‐absorption coefficients of water and active materials of the films, respectively.^(27)^ Using the values of collision kerma to water and collision kerma to films, the numerator of Eq. (3) is obtained for the ${}^{60}\text{Co}$, ${}^{137}\text{Cs}$, ${}^{192}\text{Ir}$, and ${}^{169}\text{Yb}$ sources. In the calculation of collision kerma to films, no film is present. We have assumed that the presence of the film does not affect the photon fluence spectrum and the collision kerma may be approximated to absorbed dose. In order to verify this, auxiliary simulations are carried out using the DOSRZnrc user code in which all the layers of the XRT film are modeled as cylinders. The active layer of the film is positioned at 1 cm along the transverse axis of the ${}^{169}\text{Yb}$ source. The height of the layers of the XRT film considered is 1 mm. In another similar simulation, the active layer of the film is positioned at 15 cm along the transverse axis of the ${}^{169}\text{Yb}$ source. The values of absorbed dose to active part of the XRT film obtained at 1 cm and 15 cm compare well to the values of collision kerma to the active part of XRT film obtained in the absence of the XRT film (agreement is within 0.2%).

#### C.3 Monte Carlo parameters and statistical uncertainties

The PEGS4 dataset needed for Monte Carlo calculations described above is based on the XCOM^(28)^ compilations. We set AE=0.521 MeV (kinetic energy of the electron is 0.01 MeV) and AP=0.01 MeV while generating the PEGS4 dataset, where the parameters AE and AP are the low‐energy thresholds for the production of knock‐on electrons and secondary bremsstrahlung photons, respectively. All the calculations utilized the PRESTA‐II step length and EXACT boundary crossing algorithms. In all calculations, electron range rejection technique is used to save computation time. We set ESAVE=2 MeV for this purpose.

The photon transport cut off energy PCUT is chosen at 10 keV in all calculations. In DOSRZnrc calculations, we set AE=ECUT=0.521 MeV (10 keV kinetic energy). In the FLURZnrc calculations, electrons are not transported by setting electron transport cutoff parameter ECUT=2 MeV (kinetic energy). Up to 10^9^ photon histories are simulated. The 1 σ statistical uncertainties on the calculated DOSRZnrc‐based dose values are generally within 0.3%. The 1 G statistical uncertainties on the calculated FLURZnrc‐based collision kerma values are less than 0.2%. The statistical uncertainties on the calculated values of R are less than 0.6%.

## III. RESULTS

### A. Analysis of mass energy‐absorption coefficients of films

**Figure 1:**
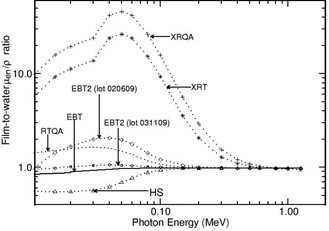
Values of mass energy‐absorption coefficients of film to water are presented for different radiochromic films as a function of photon energy in the range 10 keV−1.5 MeV.

**Figure 2:**
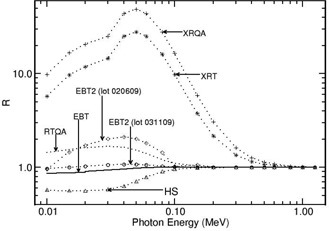
Values of the relative absorbed‐dose energy response correction R are presented for different radiochromic films as a function of photon energy in the range 10 keV−1.5 MeV The values are calculated using the mass energy‐absorption coefficients of film and water.

Figure 1 presents the mass energy‐absorption coefficient ratio of film to water for the investigated films as a function of photon energy (from 10 keV−1.25 MeV). Figure 2 presents the values of R as a function of photon energy (from 10 keV−1.25 MeV) for the investigated radiochromic films. Both figures are based on the mass energy‐absorption coefficient data from Hubbell and Selzter.^(27)^

### B. Dose ratios for ${}^{60}\text{Co}$ beam

Table 3 presents the values of film‐to‐water dose ratios for ${}^{60}\text{Co}$ beam, ${\left(\frac{D_{film}}{D_{wat}}\right)}_{60_{Co}}$ for the investigated film materials at 0.5 cm depth in water phantom. In this table, the number shown in parentheses following a value represents the absolute uncertainty on the last digit of the value with a coverage factor k=1. As shown in the table, the values of ${}^{60}\text{Co}$ beam, ${\left(\frac{D_{film}}{D_{wat}}\right)}_{60_{Co}}$ agree well with the film‐to‐water mass energy absorption coefficient ratio ${\left[\frac{(\mu_{en}/{\rho )}_{film}}{(\mu_{en}/{\rho )}_{wat}}\right]}_{60_{co}}$ at 1.25 MeV and also with the film‐to‐water electron density ratio $\frac{{\left\langle Z/A\right\rangle }_{film}}{{\left\langle Z/A\right\rangle }_{wat}}$ for all the investigated films other than XRT (deviation is 3.5% for the XRT film). Guided by the previous published work,^(15)^ we have used the values of ${\left[\frac{(\mu_{en}/{\rho )}_{film}}{(\mu_{en}/{\rho )}_{wat}}\right]}_{60_{co}}$ while calculating R.

**Table 3:** Monte Carlo‐calculated ratios of dose to film and dose to water for different radiochromic films for ${}^{60}\text{Co}$ beam are presented. Numbers in parentheses represents the absolute uncertainty on the last digit of the value with a coverage factor k=1. Active layers of these films are at a depth of 0.5 cm in a 20 cm radius by 40 cm height unit density water phantom. The ${}^{60}\text{Co}$ beam has a radius of 5.64 cm at the phantom surface. Also shown are the values of ratio of mass energy‐absorption coefficients of film to water calculated at the ${}^{60}\text{Co}$ energy (1.25 MeV) and the values of ratio of <Z/A> of film to water

*Films*	${\left(\frac{D_{film}}{D_{wat}}\right)}_{60_{Co}}$	${\left[\frac{(\mu_{en}/{\rho )}_{film}}{(\mu_{en}/{\rho )}_{wat}}\right]}_{60_{co}}$	$\frac{{\left\langle Z/A\right\rangle }_{film}}{{\left\langle Z/A\right\rangle }_{wat}}$
EBT	0.997(3)	0.983	0.982
EBT2 (lot 020609)	0.990(4)	0.981	0.969
EBT2 (lot 031109)	0.989(4)	0.982	0.971
RTQA	0.987(4)	0.976	0.975
XRT	0.985(3)	0.95	0.942
HS	0.992(3)	0.98	0.980
XRQA	0.965(3)	0.95	0.903

### C. relative absorbed‐dose energy response correction

#### B.1 ${}^{60}\text{Co}$ *brachytherapy source*

**Figure 3:**
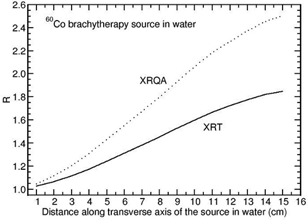
Monte Carlo‐calculated relative absorbed‐dose energy response correction R for XRT and XRQA films are shown as a function of distance along the transverse axis of the source ${}^{60}\text{Co}$ source (model Co0.A86).

Figure 3 presents the calculated values of R as a function of distance along the transverse axis of the ${}^{60}\text{Co}$ source in water phantom for the XRT and XRQA films. For the films other than XRT and XRQA, the value of R in water phantom is about unity and independent of distance for this source. The value of R increases from 1.027 to 1.845 for the XRT film and from 1.046 to 2.495 for the XRQA film when the distance is varied from 1 cm to 15 cm along the transverse axis of the ${}^{60}\text{Co}$ source (Fig. 3).

#### C.2 ${}^{137}\text{Cs}$ *brachytherapy source*

**Figure 4:**
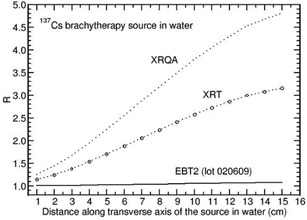
Monte Carlo‐calculated relative absorbed‐dose energy response correction R for EBT2 (lot 020609), XRT, and XRQA films are shown as a function of distance along the transverse axis of the source ${}^{137}\text{Cs}$ source (model RTR).

For ${}^{137}\text{Cs}$ source, the value of R is constant and is close to unity (with in 3%) for all the films except for EBT2 (lot 020609), XRT, and XRQA films. Figure 4 presents the values of R as a function of distance along the transverse axis of the ${}^{137}\text{Cs}$ source in water phantom for EBT2 (lot 020609), XRT, and XRQA films. The value of R increases from 1.004 to 1.075 for the EBT2 (lot 020609) film, from 1.142 to 3.155 for the XRT film, and from 1.249 to 4.816 for XRQA film when the distance is varied from 1 cm to 15 cm.

### C.3 ${}^{192}\text{Ir}$ *brachytherapy source*

**Figure 5:**
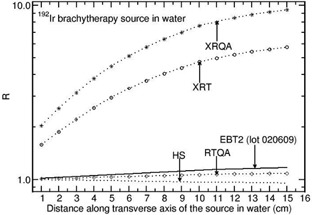
Monte Carlo‐calculated relative absorbed‐dose energy response correction R for EBT2 (lot 020609), XRT, XRQA, RTQA, and HS films are shown as a function of distance along the transverse axis of the source ${}^{192}\text{Ir}$ source (model MicroSelectron).

For ${}^{192}\text{Ir}$ source, the values of R are unity (within 1%) and are distance independent for the films EBT and EBT2 (lot03119). Figure 5 presents the values of R as a function of distance along the transverse axis of the source in water phantom for the ${}^{192}\text{Ir}$ source for the films, EBT2 (lot020609), XRT, XRQA, RTQA, and HS. The value of R increases from 1.017 to 1.169, 1.007 to 1.085, 1.586 to 5.761, and 2.035 to 9.437 for EBT2 (lot 020609), RTQA, XRT, and XRQA films, respectively, when the distance is varied from 1 cm to 15 cm along the transverse axis of the source. For the HS film, the value decreases from 0.997 to 0.954 in the above distance range.

#### C.4 ${}^{169}\text{Yb}$ *brachytherapy source*

**Figure 6:**
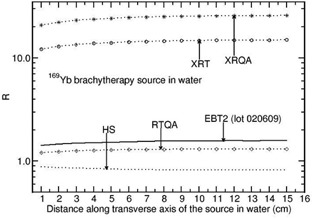
Monte Carlo‐calculated relative absorbed‐dose energy response correction R for EBT2 (lot 020609), XRT, XRQA, RTQA, and HS films are shown as a function of distance along the transverse axis of the source ${}^{169}\text{Yb}$ source (model 4140).

For ${}^{169}\text{Yb}$ source, the value of R is distant independent for the films EBT and EBT2 (lot 031109). For the EBT film, the value is about unity (within 1%) and about 1.040 for EBT2 (lot 031109). Figure 6 presents the values of R for the ${}^{169}\text{Yb}$ (model 4140) source as a function of distance along the transverse axis of the source in water phantom for the films, EBT2 (lot020609), XRT, XRQA, RTQA, and HS. The value of R increases from 1.416 to 1.576, 1.215 to 1.313, 12.200 to 14.980, and 20.872 to 25.838 for EBT2 (lot 020609), RTQA, XRT, and XRQA films, respectively, when the distance is varied from 1 cm to 15 cm. However, the value of R decreases from 0.881 to 0.820 for the HS film in the abovementioned distance range.

### D. Influence of solid phantom materials on R

The values of R obtained in the solid phantom materials PMMA, polystyrene, and water are designated as $R_{\text{PMMA}},R_{\text{Poly}}$, and $R_{\text{wat}}$, respectively. For ${}^{60}\text{Co}$, ${}^{137}\text{Cs}$, and ${}^{192}\text{Ir}$ sources, the ratios $R_{\text{PMMA}}/R_{\text{wat}}$ and $R_{\text{Poly}}/R_{\text{wat}}$ are not significantly different from unity (within 1% for ${}^{60}\text{Co}$, 3% for ${}^{137}\text{Cs}$ and for ${}^{192}\text{Ir}$) at all distances for all the films except for the XRT and XRQA films. For the XRT and XRQA films, the above‐mentioned ratios increase with distance. For ${}^{60}\text{Co}$ source, the values of $R_{\text{PMMA}}/R_{\text{wat}}$ are 1.202 and 1.265, whereas the values of $R_{\text{Poly}}/R_{\text{wat}}$ are 1.140 and 1.185, respectively, for XRT and XRQA films at a distance of 15 cm from the source. For ${}^{137}\text{Cs}$ source, the values of $R_{\text{PMMA}}/R_{\text{wat}}$ are 1.260 and 1.302, whereas the values of $R_{\text{Poly}}/R_{\text{wat}}$ are 1.191 and 1.223, respectively, for XRT and XRQA films at a distance of 15 cm from the source. For ${}^{192}\text{Ir}$ source, the values of $R_{\text{PMMA}}/R_{\text{wat}}$ are 1.240 and 1.261, whereas the values of $R_{\text{Poly}}/R_{\text{wat}}$ are 1199 and 1.217, respectively, for XRT and XRQA films at a distance of 15 cm from the source. A similar comparison for ${}^{169}\text{Yb}$ source shows that the ratios $R_{\text{PMMA}}/R_{\text{wat}}$ and $R_{\text{Poly}}/R_{\text{wat}}$ are about unity for the films EBT and EBT2 (lot 031109). For the remaining films, the ratios are distant dependent. For example, the values of $R_{\text{Poly}}/R_{\text{wat}}$ are up to 8% to 9% higher than unity for the films EBT2 (lot 020609), RTQA, XRT, and XRQA at 15 cm. For these films, the values of $R_{\text{PMMA}}/R_{\text{wat}}$ are up to 5% to 8% higher than unity at 15 cm.

## IV. DISCUSSION

$Z_{\text{eff}}$ values of active materials the films and the fluence‐weighted mean energy (hereafter referred to as mean energy) of photons in the phantoms play a role on the values of R. The mean energy calculated using the FLURZnrc^(16)^ user code decreases as the distance along the transverse axis of the sources increases. For example, for the ${}^{60}\text{Co}$ source, the mean energy in water phantom decreases from 1.150 MeV to 0.520 MeV when the distance is increased from 1 to 15 cm. In this distance range, the mean energy decreases from 0.566 MeV to 0.260 MeV, 0.326 MeV to 0.160 MeV, and 0.103 MeV to 0.082 MeV for the ${}^{137}\text{Cs}$, ${}^{192}\text{Ir}$, and ${}^{169}\text{Yb}$ sources, respectively. The decrease in the mean energy affects the value of R of the films that have higher or smaller $Z_{\text{eff}}$ values (active materials of the XRT and XRQA films have higher $Z_{\text{eff}}$ values, whereas HS film has smaller $Z_{\text{eff}}$ value). The HS film does not show energy dependence for the ${}^{60}\text{Co}$ at all distances. For this film the value of R is 0.98 at 15 cm from the ${}^{137}\text{Cs}$ source. For the ${}^{192}\text{Ir}$ and ${}^{169}\text{Yb}$ sources, the value of R decreases as the distance increases (see Figs. 5 and 6).

The energy degradation of photons in the PMMA and polystyrene phantoms is different when compared to the water phantom. This affects the values of R, and the change in R is significant for the XRT and XRQA films for the ${}^{60}\text{Co}$, ${}^{137}\text{Cs}$, and ${}^{192}\text{Ir}$ sources (see Results section D above). In the distance range of 1‐15 cm, the mean energy decreases from 1.134 MeV to 0.455 MeV, 0.557 MeV to 0.228 MeV, 0.320 MeV to 0.142 MeV, and 0.101 MeV to 0.074 MeV, respectively, for the ${}^{60}\text{Co}$, ${}^{137}\text{Cs}$, ${}^{192}\text{Ir}$, and ${}^{169}\text{Yb}$ sources in the PMMA phantom. The corresponding values in the polystyrene phantom are 1.146 MeV−0.486 MeV, 0.563 MeV−0.239 MeV. 0.324 MeV−0.148 MeV, and 0.102 MeV−0.069 MeV.

The values of R calculated in the present study are applicable along the transverse axis of the sources (1‐15 cm). As the investigated sources exhibit anisotropy in the dose profiles, there is a possibility that the R values may exhibit angular dependence for some films, which is beyond the scope of present study.

## V. CONCLUSIONS

Relative absorbed‐dose energy response correction R for various radiochromic films in water phantom is calculated by the use of the Monte Carlo‐based EGSnrc code system for high energy brachytherapy sources ${}^{60}\text{Co}$, ${}^{137}\text{Cs}$, ${}^{192}\text{Ir}$, and ${}^{169}\text{Yb}$. For ${}^{60}\text{Co}$ source, the value of R is about unity and is independent of distance in water phantom for the films other than XRT and XRQA. The XRT and XRQA films showed distance‐dependent R values for this source. In the case of ${}^{137}\text{Cs}$ and ${}^{192}\text{Ir}$ sources, XRT, XRQA, EBT2 (lot 031109), and HS films showed distant‐dependent R values, and the rest showed no energy dependence. HS film showed R values less than unity by about 5%, whereas the other films showed R values higher than unity for these sources. In the case of ${}^{169}\text{Yb}$ source, the EBT film showed no energy dependence and EBT2 film (lot 031109) showed a distance‐independent R value of 1.041. The rest showed distance‐dependent R values (increases with distance for the films other than HS). The solid phantoms, such as PMMA and polystyrene, enhance the R values for some film materials when compared the same in water phantom.

## ACKNOWLEDGMENTS

The authors wish to thank Dr. D. N. Sharma, Director, Health, Safety and Environment Group, Bhabha Atomic Research Centre (BARC), for his encouragement and support throughout this project.
